# MDM2 SNP309 polymorphism contributes to endometrial cancer susceptibility: evidence from a meta-analysis

**DOI:** 10.1186/1756-9966-32-85

**Published:** 2013-11-03

**Authors:** Qiliu Peng, Cuiju Mo, Aiping Qin, Xianjun Lao, Zhiping Chen, Jingzhe Sui, Junrong Wu, Limin Zhai, Shi Yang, Xue Qin, Shan Li

**Affiliations:** 1 Department of Clinical Laboratory, First Affiliated Hospital of Guangxi Medical University, Nanning, Guangxi, China; 2 Department of Obstetrics and Gynecology and Reproductive center, First Affiliated Hospital of Guangxi Medical University, Nanning, Guangxi, China; 3 Department of Occupational Health and Environmental Health, School of Public Health at Guangxi Medical University, Nanning, Guangxi, China

**Keywords:** Endometrial cancer, MDM2, SNP309, Meta-analysis

## Abstract

**Objective:**

The SNP309 polymorphism (T-G) in the promoter of MDM2 gene has been reported to be associated with enhanced MDM2 expression and tumor development. Studies investigating the association between MDM2 SNP309 polymorphism and endometrial cancer risk reported conflicting results. We performed a meta-analysis of all available studies to explore this association.

**Methods:**

All studies published up to August 2013 on the association between MDM2 SNP309 polymorphism and endometrial cancer risk were identified by searching electronic databases PubMed, Web of Science, EMBASE, and Chinese Biomedical Literature database (CBM). The association between the MDM2 SNP309 polymorphism and endometrial cancer risk was assessed by odds ratios (ORs) together with their 95% confidence intervals (CIs).

**Results:**

Eight case–control studies with 2069 endometrial cancer cases and 4546 controls were identified. Overall, significant increase of endometrial cancer risk was found when all studies were pooled in the meta-analysis (GG vs. TT: OR = 1.464, 95% CI 1.246–1.721, *P* < 0.001; GG vs. TG + TT: OR = 1.726, 95% CI 1.251–2.380, *P* = 0.001; GG + TG vs. TT: OR = 1.169, 95% CI 1.048–1.304, *P* = 0.005). In subgroup analysis by ethnicity and HWE in controls, significant increase of endometrial cancer risks were observed in Caucasians and studies consistent with HWE. In subgroup analysis according to study quality, significant associations were observed in both high quality studies and low quality studies.

**Conclusions:**

This meta-analysis suggests that MDM2 SNP309 polymorphism contributes to endometrial cancer susceptibility, especially in Caucasian populations. Further large and well-designed studies are needed to confirm this association.

## Introduction

Endometrial cancer is one of the most common gynecologic cancers in developed countries [1,2]. Although its incidence rates are up to ten times higher in industrialized countries when compared to Asia or Africa, its prevalence has also been increasing in developing countries during the last decades [2]. As with all solid tumors, endometrial cancer is a heterogeneous disease with complex genetic and environmental influences. It has been suggested that environmental risk factors such as obesity and overexposure to endogenous or exogenous hormones may be involved in the pathogenesis of endometrial cancer [3,4]. In addition, predisposition to endometrial cancer is mediated by genetic factors including both germinal and somatic alterations as well as genetic polymorphisms [5,6].

The murine double minute-2 (MDM2) is a key negative regulator of the P53 tumor suppressor pathway which has been suggested to be implicated in a variety of cancers [7]. Evidence shows that MDM2 can bind directly to P53 protein and inhibit its activity, thus resulting in its degradation via the ubiquitination pathway [8]. A single nucleotide polymorphism (SNP) in the promoter region of MDM2, SNP T309G (rs2279744), has been identified and was demonstrated to up-regulate the expression of MDM2 via a greater affinity for the SP1 transcription factor. Consequently, individuals carrying the GG genotype of the MDM2 SNP309 polymorphism were found to have higher MDM2 levels, which led to attenuation of the TP53 pathway and acceleration of tumor formation in humans [9]. It was reported that the increase in MDM2 results in direct inhibition of p53 transcriptional activity, enabling damaged cells to escape the cell-cycle checkpoint and become carcinogenic [10]. Hence, it is biologically reasonable to hypothesize a potential relationship between the MDM2 SNP309 polymorphism and endometrial cancer risk.

Over the last two decades, a number of molecular epidemiological studies have been conducted to investigate the association between the MDM2 SNP309 polymorphism and endometrial cancer risk, but the results remain inconsistent. Several studies have previously suggested that the MDM2 SNP309 polymorphism was associated with an increased risk of endometrial cancer [11-13]. However, other studies have failed to confirm such an association [14,15]. In addition, a meta-analysis including six studies by Li et al. [16] found that the GG genotype of MDM2 SNP309 polymorphism was significantly associated with the increased endometrial cancer risk. However, they included two studies containing overlapping data [13,17] in their meta-analysis, which might make their conclusions questionable. As new studies emerge [15,18,19], to provide the most comprehensive assessment of the associations between the MDM2 SNP309 polymorphism and endometrial cancer risk, we performed a meta-analysis of all available studies.

## Materials and methods

### Search strategy

We conducted a comprehensive literature search in PubMed, Web of Science, EMBASE, and Chinese Biomedical Literature (CBM) databases up to August 01, 2013 using the following search strategy: (“endometrial cancer”) and (“Murine double minute 2”, or “MDM2”). There was no restriction on time period, sample size, population, language, or type of report. All eligible studies were retrieved and their references were checked for other relevant studies. The literature retrieval was performed in duplication by two independent investigators (Qiliu Peng and Cuiju Mo).

### Inclusion and exclusion criteria

Studies included in the meta-analysis were required to meet the following criteria: (1) Case–control studies which evaluated the association between MDM2 SNP309 polymorphism and endometrial cancer risk; (2) used an unrelated case–control design; (3) had an odds ratio (OR) with 95% confidence interval (CI) or other available data for estimating OR (95% CI); and (4) the control population did not contain malignant tumor patients. Conference abstracts, case reports, editorials, review articles, and letters were excluded. When multiple publications reported on the same or overlapping data, we chose the most recent or largest population. When a study reported the results on different subpopulations, we treated it as separate studies in the meta-analysis.

### Data extraction

Two reviewers (Qiliu Peng and Cuiju Mo) independently reviewed and extracted data from all eligible studies. Data extracted from eligible studies included the first author, year of publication, country of origin, ethnicity, genotyping method, matching criteria, source of control, endometrial cancer confirmation criteria, total number of cases and controls and genotype frequencies of cases and controls. Ethnic backgrounds were categorized as Caucasian and Asian. To ensure the accuracy of the extracted information, the two investigators checked the data extraction results and reached consensus on all of the data extracted.

### Methodological quality assessment

Methodological quality was independently assessed by two reviewers (Qiliu Peng and Cuiju Mo), according to a set of predefined criteria (Additional file 1: Table S1) based on the scale of Thakkinstian et al. [20]. The revised criteria cover the representativeness of cases, the credibility of controls, ascertainment of endometrial cancer, genotyping examination, Hardy-Weinberg equilibrium (HWE) in the control population, and association assessment. Disagreements were resolved by consensus. Scores ranged from 0 (lowest) to 12 (highest). Articles with scores less than 8 were considered “low-quality” studies, whereas those with scores equal to or higher than 8 were considered “high-quality” studies.

### Statistical analysis

The strength of the association between MDM2 SNP309 polymorphism and endometrial cancer risk was assessed by odds ratios (ORs) with 95% confidence intervals (CIs). The significance of the pooled OR was determined by Z test and a *p* value of less than 0.05 was considered significant. The association of MDM2 SNP309 polymorphism with endometrial cancer risk was assessed using additive models (GG vs. TT and TG vs. TT), recessive model (GG vs. TG + TT), and dominant model (GG + TG vs. TT).

The χ^2^ based *Q* test and *I*^*2*^ statistics were used to assess the heterogeneity among studies [21,22]. If the result of the *Q* test was *P*_*Q*_ < 0.1 or *I*^*2*^ ≥ 50%, indicating the presence of heterogeneity, a random-effects model (the DerSimonian and Laird method) was used to estimate the summary ORs [23]; otherwise, when the result of the *Q* test was *P*_*Q*_ ≥ 0.1 and *I*^*2*^ < 50%, indicating the absence of heterogeneity, the fixed-effects model (the Mantel–Haenszel method) was used [24]. To explore the sources of heterogeneity among studies, we performed logistic metaregression and subgroup analyses. The following study characteristics were included as covariates in the metaregression analysis: genotyping methods (PCR-RFLP vs. not PCR-RFLP), ethnicity (Caucasians vs. Asians), source of controls (Hospital-based vs. Population-based), quality scores (High-quality vs. Low-quality), HWE status (Yes vs. No), and endometrial cancer confirmation (pathologically or histologically confirmed vs. other diagnosis criteria). Subgroup analyses were conducted by ethnicity, study quality, and HWE in controls. Galbraith plots analysis was performed for further exploration of the heterogeneity.

Sensitivity analysis was performed by sequential omission of individual studies. Publication bias was evaluated using a funnel plot and Egger’s regression asymmetry test [25]. The distribution of the genotypes in the control population was tested for HWE using a goodness-of-fit χ^2^ test. All analyses were performed using Stata software, version 12.0 (Stata Corp., College Station, TX).

## Result

### Study characteristics

With our search criterion, 35 individual records were found, but only ten full-text publications were preliminarily identified for further detailed evaluation. According to the exclusion criteria, three publications were excluded including one publication containing overlapped data [17], one was a meta-analysis [16], and one was a letter [26]. Manual search of references cited in the published studies did not reveal any additional articles. As a result, a total of seven relevant studies met the inclusion criteria for the meta-analysis [11-15,18,19]. Among them, one of the eligible studies contained data on two different ethnic groups [12], and we treated it independently. Therefore, a total of eight separate comparisons including 2069 endometrial cancer cases and 4546 controls were finally included in our meta-analysis. The main characteristics of the studies are presented in Table 1. Of all the eligible studies, six were conducted in Caucasian populations, and two were in Asians. Four studies were population–based and four were hospital–based studies. All studies used validated methods including PCR-RFLP, TaqMan assay to genotype the MDM2 SNP309 polymorphism. The endometrial cancer cases were histologically or pathologically confirmed in five of the eligible studies. The genotype distribution of the controls in one study was not consistent with HWE [13].

**Table 1:** Characteristics of studies included in this meta-analysis

**First author (Year)**	**Country**	**Ethnicity**	**Sample size (case/control)**	**Genotyping methods**	**Matching criteria**	**Source of control**	**EC confirmation**	**Quality scores**	**HWE (*P *** **value)**
Walsh [11]	America	Caucasian	73/79	PCR-RFLP	NA	HB	NA	5.5	0.650
Terry NHS [12]	America	Caucasian	394/948	PCR-RFLP	Age, menopausal status	PB	PC	11	0.642
Terry WHS [12]	America	Caucasian	122/368	PCR-RFLP	Age, menopausal status	PB	PC	11	0.180
Ashton 2009 [14]	Australia	Caucasian	191/291	TaqMan Assay	Age, gender	PB	HC	9	0.493
Nunobiki [13]	Japan	Asian	102/95	PCR-RFLP	NA	HB	HC	5	0.018
Zajac [18]	Poland	Caucasian	152/100	PCR-RFLP	NA	HB	HC	6.25	0.701
Knappskog [19]	Norway	Caucasian	910/2465	TaqMan Assay	NA	HB	NA	8	0.406
Yoneda [15]	Japan	Asian	125/200	PCR-RFLP	NA	PB	NA	9	0.910

EC, Endometrial cancer; HC, Histologically confirmed; PC, Pathologically confirmed; NA, Not available; PB, Population–based; HB, Hospital–based;

HWE, Hardy–Weinberg equilibrium in control population; PCR–RFLP, Polymerase chain reaction-restriction fragment length polymorphism.

### Meta-analysis

**Figure 1:**
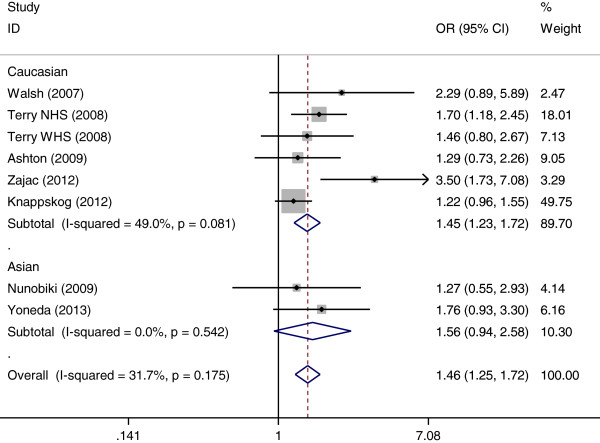
Forest plots of MDM2 SNP309 polymorphism and endometrial cancer risk in subgroup analysis by ethnicity using a fixed-effect model (additive model GG vs. TT).

**Figure 2:**
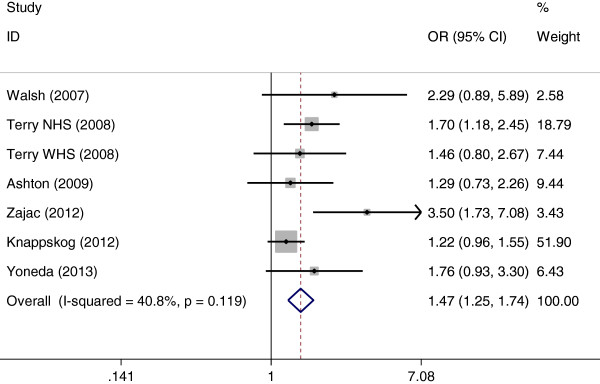
Forest plots of MDM2 SNP309 polymorphism and endometrial cancer risk in studies consistent with HWE using a fixed-effect model (additive model GG vs. TT).

The results of the association between MDM2 SNP309 polymorphism and endometrial cancer risk were shown in Table 2. Overall, significant elevated endometrial cancer risk was found when all studies were pooled into the meta-analysis (GG vs. TT: OR = 1.464, 95% CI 1.246–1.721, *P* < 0.001, Figure 1; GG vs. TG + TT: OR = 1.726, 95% CI 1.251–2.380, *P* = 0.001; GG + TG vs. TT: OR = 1.169, 95% CI 1.048–1.304, *P* = 0.005). In subgroup analysis by ethnicity, significant increased endometrial cancer risk was found in Caucasians (GG vs. TT: OR = 1.602, 95% CI 1.208–2.125, *P* = 0.001; GG vs. TG + TT: OR = 1.748, 95% CI 1.161–2.632, *P* = 0.007; GG + TG vs. TT: OR = 1.173, 95% CI 1.047–1.315, *P* = 0.006) but not in Asians. In stratified analysis by HWE in controls, significant increased endometrial cancer risk was also observed in studies consistent with HWE (GG vs. TT: OR = 1.473, 95% CI 1.249–1.737, *P* < 0.001, Figure 2; GG vs. TG + TT: OR = 1.471, 95% CI 1.267–1.707, *P* < 0.001; GG + TG vs. TT: OR = 1.184, 95% CI 1.060–1.323, *P* = 0.003). When stratified by study quality, significant associations were found in both high quality studies and low quality studies.

**Table 2:** Meta-analysis of MDM2 309 T/G polymorphism and endometrial cancer risk

**Analysis**	**No. of studies**	**Homozygote (GG vs. TT)**		**Heterozygote (TG vs. TT)**		**Dominant model (GG + TG vs. TT)**		**Recessive model (GG vs. TG + TT)**	
		**OR (95% CI)**	***P/P***_**Q**_	**OR (95% CI)**	***P/P***_**Q**_	**OR (95% CI)**	***P/P***_**Q**_	**OR (95% CI)**	***P/P***_**Q**_
Overall	8	1.464 (1.246-1.721)	0.000/0.175	1.073 (0.955-1.205)	0.238/0.312	1.169 (1.048-1.304)	0.005/0.759	1.726 (1.251-2.380)	0.001/0.000
Ethnicity									
Caucasian	6	1.453 (1.225-1.724)	0.000/0.181	1.084 (0.960-1.223)	0.192/0.521	1.173 (1.047-1.315)	0.006/0.900	1.748 (1.161-2.632)	0.007/0.000
Asian	2	1.560 (0.943-2.581)	0.083/0.542	0.855 (0.358-2.038)	0.723/0.156	1.047 (0.531-2.064)	0.894/0.113	0.981 (0.813-1.525)	0.212/0.494
Study quality									
High quality	5	1.376 (1.157-1.637)	0.000/0.569	1.120 (0.992-1.264)	0.068/0.883	1.174 (1.047-1.316)	0.006/0.929	1.495 (1.293-1.728)	0.002/0.368
Low quality	3	2.264 (1.421-3.607)	0.001/0.191	0.748 (0.428-1.023)	0.121/0.705	1.118 (0.766-1.631)	0.563/0.195	3.124 (2.146-4.548)	0.000/0.130
HWE in controls									
Yes	7	1.473 (1.249-1.737)	0.000/0.119	1.093 (0.971-1.230)	0.141/0.601	1.184 (1.060-1.323)	0.003/0.907	1.471 (1.267-1.707)	0.000/0.000
No	1	1.268 (0.549-2.928)	0.579/—	0.528 (0.254-1.100)	0.088/—	0.708 (0.353-1.421)	0.332/—	1.830 (0.974-3.830)	0.067/—

*P*_Q_ P values of Q-test for heterogeneity test. OR, odds ratio; CI, confidence intervals; HWE, Hardy–Weinberg equilibrium.

### Test of heterogeneity

**Figure 3:**
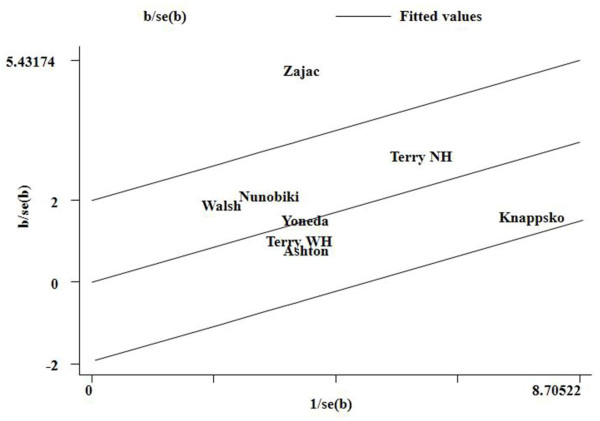
**Galbraith plots of MDM2 SNP309 polymorphism and endometrial cancer risk in the overall populations (Recessive model GG vs. TG + TT).** The study of Zajac et al. was spotted as outlier.

Statistical significant heterogeneity among studies was observed in the association analysis between the MDM2 SNP309 polymorphism and endometrial cancer risk in the overall populations (GG vs. GT + TT: *P*_*Q*_ < 0.001; Table 2). To explore the sources of heterogeneity, we performed metaregression and subgroup analyses. Metaregression analysis of data showed that the ethnicity, study quality, and HWE status were the sources which contributed to heterogeneity. Subsequently, we performed subgroup analyses stratified by ethnicity, study quality, and HWE status. However, heterogeneity still existed among Caucasians (GG vs. GT + TT: *P*_*Q*_ < 0.001), and studies consistent with HWE (GG vs. GT + TT: *P*_*Q*_ < 0.001). To further investigate the heterogeneity, we performed Galbraith plots analysis to identify the outliers which might contribute to the heterogeneity. Our results showed that the study Zajac et al. [18] was the outlier in the overall populations (Figure 3). All *I*^*2*^ values decreased obviously and *P*_*Q*_ values were greater than 0.10 after excluding the study Zajac et al. [18] in the overall populations (GG vs. GT + TT: *P*_*Q*_ = 0.241), Caucasians (GG vs. GT + TT: *P*_*Q*_ = 0.179), and studies consistent with HWE (GG vs. GT + TT: *P*_*Q*_ = 0.260). However, the significance of the summary ORs for MDM2 SNP309 polymorphism in the overall population and subgroup analyses were not influenced by omitting the study by Zajac et al. [18].

### Sensitivity analysis

Sensitivity analysis was performed to assess the influence of each individual study on the pooled OR by sequential removal of individual studies. The results suggested that no individual study significantly affected the pooled ORs, indicating that our results were robust and reliable.

### Publication bias

**Figure 4:**
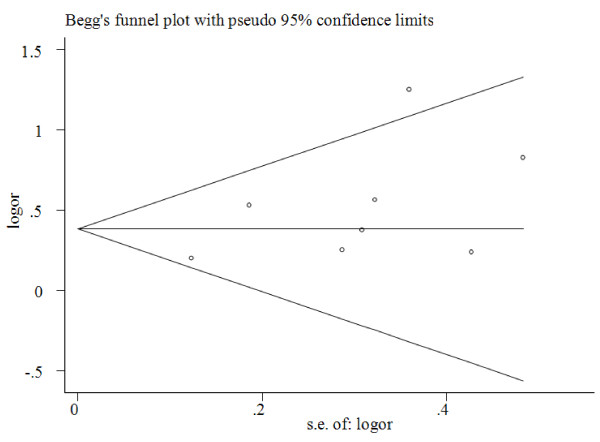
Funnel plots for publication bias of the meta-analysis on the association between MDM2 SNP309 polymorphism and endometrial cancer risk of the overall populations (additive model GG versus TT).

Begg’s funnel plot and Egger’s test were performed to access the publication bias of literatures in this meta-analysis. The shapes of Funnel plot did not reveal obvious evidence of asymmetry, and all the p values of Egger’s tests were more than 0.05, providing statistical evidence of the funnel plots’ symmetry (Figure 4). Thus, the results above suggested that publication bias was not evident in this meta-analysis.

## Discussion

It has been shown that estrogen signaling affect MDM2 expression levels through an interaction of estrogen receptor (ER) with a region of the MDM2 promoter [27,28]. SNP309 was found in the region of the promoter where ER binds and leads to transcription of the MDM2 gene [29]. Furthermore, the G allele of SNP309 increases the affinity of the MDM2 promoter for the transcription factor Sp1 [27]. Sp1 is a co-transcriptional activator of many hormone receptors, including ER [30] and is known to participate in estrogen-mediated gene transcription [31,32]. The effects of overexpressed MDM2 may be enhanced by ER interactions with Sp1 [33]. These observations lend further biological plausibility to the association between MDM2 SNP309 and the development of endometrial cancer, a highly estrogen-dependent neoplasm. To date, a number of epidemiological studies have evaluated the association between MDM2 SNP309 polymorphism and endometrial cancer risk, but the results remain inconclusive. To derive a more precise estimation of relationship, we performed this meta-analysis. Our meta-analysis based on eight case–control studies suggested that the MDM2 SNP309 polymorphism contributes to increased endometrial cancer susceptibility.

In the subgroup analysis by ethnicity, significantly increased endometrial cancer risk was found in Caucasians. However, no significant association was found in Asians. It might not be uncommon for the same polymorphism playing different roles in cancer susceptibility among different ethnic populations, because cancer is a complicated multi-genetic disease, and different genetic backgrounds may contribute to the discrepancy. Nevertheless, owing to the limited number of relevant studies among Asian populations included in this meta-analysis, the observed negative association between MDM2 SNP309 polymorphism and endometrial cancer risk in Asians is likely to be caused by chance because study with small sample sizes may have insufficient statistical power to detect a slight effect or may have generated a fluctuated risk estimate. Currently there were only two studies [13,15] on MDM2 SNP309 polymorphism and endometrial cancer risk in Asian populations, and the genotype distributions in the control population of one study [13] was deviate from HWE. Therefore, the negative results of the Asian population should be interpreted with caution.

To clarify an association between genetic polymorphisms and cancer risk, the quality of the study design is of great importance. In addition to controls that should be in HWE, strict definitions of the study population, appropriate materials used to assess genotype as well as sufficient statistical power are required. Of the eigh eligible studies, three were considered as low quality studies [11,13,18] and 5 were considered as high quality studies [12,14,15,19]. When stratified according to the quality of the articles, we found that the MDM2 SNP309 polymorphism was associated with elevated endometrial cancer risk in both high and low quality studies in additive model (CC vs. CG) and recessive model (GG vs. TG + TT). Interestingly, similarly elevated risks were found in high quality studies, but not in low quality studies in the dominant model (GG + TG vs. TT). Several possibilities exist which may explain this finding, such as selection bias and recall bias. Genotyping methods without quality control in low quality studies should be considered when deciphering these inconsistent results, which reinforces that the importance of precise methodologically design is of great value in case–control studies.

It seemed that selection bias could have played a role because the genotype distribution of the MDM2 SNP309 polymorphism among control subjects disobeyed the law of HWE in one of the included studies [13]. It is widely believed that deviation from HWE may be as a result of genetic reasons including non-random mating, or the alleles reflect recent mutations that have not reached equilibrium, as well as methodological reasons including biased selection of subjects from the population or genotyping errors [34,35]. Because of the reasons of disequilibrium, the results of genetic association studies might be spurious if the distribution of genotypes in the control groups were not in HWE [36,37]. Hence, we carried out subgroup analysis by HWE in controls. When excluding the study that was not in HWE, the results were persistent and robust, suggesting that this factor probably had little effect on the overall estimates.

Heterogeneity is a potential problem when interpreting the results of a meta-analysis, and finding the sources of heterogeneity is one of the most important goals of meta-analysis [38]. In the present meta-analysis, significant between-study heterogeneity in the pooled analyses of total eligible studies was observed in recessive model GG vs. TG + TT (The *P*_*Q*_ value was less than 0.001). To find the sources of heterogeneity, we performed metaregression and subgroup analyses. Metaregression analysis of data showed that the ethnicity, study quality, and HWE status were the sources of heterogeneity. Subgroup analyses stratified by ethnicity, study quality, and HWE status showed that the heterogeneity was still significant in Caucasians and studies consistent with HWE. To further investigate the heterogeneity, Galbraith plots analysis was performed to identify the outliers which might contribute most to the heterogeneity. Our results showed that the study of Zajac et al. [18] was the outlier of recessive model GG vs. TG + TT in the overall population, Caucasians, and studies consistent with HWE. All *I*^*2*^ values decreased lower than 50% and *P*_*Q*_ values were larger than 0.10 after excluding the studies of Zajac et al. [18] in the recessive model GG vs. TG + TT in the overall population, Caucasians, and studies consistent with HWE. However, the summary ORs for the MDM2 SNP309 polymorphism in recessive model GG vs. TG + TT in the overall population, Caucasians, and studies consistent with HWE were not material change by omitting this study, indicating that our results were robust and reliable. The results indicated that the study of Zajac et al. [18] might be the major source of the heterogeneity in the meta-analysis.

Some limitations of this meta-analysis should be addressed. First, in subgroup analysis by ethnicity, the included studies regarded only Asians and Caucasians. Data concerning other ethnicities such as Africans were not found. Thus, additional studies are warranted to evaluate the effect of this functional polymorphism on endometrial cancer risk in different ethnicities, especially in Africans. Second, our results were based on unadjusted estimates. We did not perform the analysis adjusted for other covariates such as age, obesity, drinking and smoking status, menopausal status, use of contraceptives, environment factors, and so on, because of the unavailable original data of the eligible studies.

In conclusion, this meta-analysis suggests that the MDM2 SNP309 polymorphism may be associated with increased risk of developing endometrial cancer particularly among Caucasians. However, it is necessary to conduct large sample studies using standardized unbiased genotyping methods, homogeneous endometrial cancer patients, and well-matched controls. Moreover, gene–gene and gene–environment interactions should also be considered in the analysis. Such studies taking these factors into account may eventually lead to our better, comprehensive understanding of the association between the MDM2 SNP309 polymorphism and endometrial cancer risk.

## Consent

Written informed consent was obtained from the patient for the publication of this report and any accompanying images.

## Abbreviations

HWE: Hardy–Weinberg equilibrium; MDM2: Murine double minute 2; SNP: Single nucleotide polymorphism; OR: Odds ratio; CI: Confidence interval.

## Competing interests

The authors do not have any potential competing interests.

## Authors’ contributions

PQL, QAP, LXJ and MCJ conceived and designed the study, CZP, SJZ, WJR, ZLM, YS, QX, and LS participated in selecting study, extracting data, performing the statistical analysis and drafting the manuscript. PQL has been involved in revising the manuscript critically for important intellectual content. All authors read and approved the final manuscript.

## Supplementary Material

Additional file 1: Table S1 Scale for Quality Assessment. Click here for file
