# Supplementary material for: MDM2 SNP309 polymorphism contributes to endometrial cancer susceptibility: evidence from a meta-analysis
Source: J Exp Clin Cancer Res. 2013 Nov 3;32(1):85. doi: 10.1186/1756-9966-32-85 (PMC4029393; doi:10.1186/1756-9966-32-85)
Supplement: Additional file 1: Table S1 — Scale for Quality Assessment. [file 1756-9966-32-85-S1.doc]

Additional file 1: Table S1. Scale for Quality Assessment

| Criteria | Score |
| --- | --- |
| Representativeness of cases |  |
| Selected from population or cancer registry | 2 |
| Selected from any gynaecology /surgery service | 1 |
| Selected without clearly defined sampling frame or with extensive inclusion/exclusion criteria | 0 |
| Credibility of controls |  |
| Population- or neighbor- based | 3 |
| Blood donors or volunteers | 2 |
| Hospital-based (cancer-free patients) | 1 |
| Healthy volunteers, but without total description | 0.5 |
| Gynaecology patients | 0.25 |
| Not described | 0 |
| Ascertainment of endometrial cancer |  |
| Histological or pathological confirmation | 2 |
| Diagnosis of endometrial cancer by patient medical record | 1 |
| Not described | 0 |
| Genotyping examination |  |
| Genotyping done under ‘‘blinded’’ condition | 1 |
| Unblinded or not mentioned | 0 |
| Hardy-Weinberg equilibrium |  |
| Hardy-Weinberg equilibrium in controls | 2 |
| Hardy-Weinberg disequilibrium in controls | 1 |
| No checking for Hardy-Weinberg disequilibrium | 0 |
| Association assessment |  |
| Assess association between genotypes and endometrial cancer with appropriate statistics and adjustment for confounders | 2 |
| Assess association between genotypes and endometrial cancer with appropriate statistics without adjustment for confounders | 1 |
| Inappropriate statistics used | 0 |
